# Preparing current and emerging health leaders: An innovative competency-based framework for health diplomacy

**DOI:** 10.1371/journal.pgph.0004974

**Published:** 2026-09-15

**Authors:** Esperanza Martinez, Ellen Rosskam, Garry Aslanyan, Lyndsay Baines, Ryuichi Komatsu, Nikica Darabos, Brian Li Han Wong, Nina Viberg, Mariko Hososawa, Ren Minghui, Magda Robalo Correia e Silva, Anders Nordstrom

**Affiliations:** 1 College of Law, Governance and Policy, Australian National University, Canberra, Australia; 2 Global Health Centre, Geneva Graduate Institute, Geneva, Switzerland; 3 Center for Social Epidemiology, Los Angeles, United States of America; 4 Dalla Lana School of Public Health, University of Toronto, Toronto, Canada; 5 UNICEF/UNDP/World Bank/WHO Special Programme for Research and Training in Tropical Diseases, Geneva, Switzerland; 6 Wolfson College, University of Oxford, United Kingdom; 7 Interfaculty Initiative in Planetary Health, Nagasaki University, Nagasaki, Japan; 8 School of Tropical Medicine and Global Health, Nagasaki University, Nagasaki, Japan; 9 Navigo Partners, LLC, Japan; 10 Faculty of Economics, Business and Tourism and the Faculty of Medicine of the University of Split, Croatia; 11 Department of International Health, Care and Public Health Research Institute, Maastricht University, Maastricht, Netherlands; 12 Department of Global Public Health and Center of Excellence for Sustainable Health, Karolinska Institutet, Sweden; 13 Institute for Global Health Policy Research, Bureau of Global Health Cooperation, Japan Institution for Health Security, Japan; 14 Department of Global Health, School of Public Health, Peking University, China; 15 Institute for Global Health, Peking University, China; 16 China Center for Health Development Studies, Peking University, China; 17 Institute for Global Health and Development, Guinea-Bissau; 18 Department of Global Public Health Karolinska Institutet, Stockholm, Sweden; 19 Center for resilient health, Stockholm School of Economics, Stockholm, Sweden; National Center for Chronic and Noncommunicable Disease Control and Prevention, Chinese Center for Disease Control and Prevention, CHINA

## Abstract

Health diplomacy is increasingly recognized as critical domain of practice for advancing global health equity, navigating political complexity, and fostering cooperation across sectors and borders. Yet formal training opportunities remain limited worldwide, with existing programs differing in length, depth and content, and lacking a shared core curriculum. To address these persistent gaps, the Health Diplomacy Institutional Network (HDIN) developed a globally informed, competency-based training framework that contributes to the professionalization of the field by offering a shared foundation for capacity building in health diplomacy. Co-developed by global experts and practitioners, the framework identifies five key competencies – global health knowledge, political and diplomatic acumen, strategic leadership, cross-cultural communication, and advocacy and coalition-building – each articulated across four progressive levels of mastery, from basic to expert. This structure enables institutions to align training content with learners’ roles, experience levels, and contexts. The framework’s flexible, outcomes-based design allows for adaptation across institutional missions, educational formats, and geopolitical settings. Accompanied by practical implementation tools, the HDIN Training Framework supports high-quality, context-responsive capacity-building for academic institutions, governments, NGOs, and multilateral organizations. It aims to equip a health diplomacy workforce capable of advancing health priorities amid intensifying global interdependence and uncertainty.

## Introduction: The imperative for health diplomacy

The COVID-19 pandemic left a profound mark on humanity, costing millions of lives and triggering global disruptions in education, employment, and economic stability [1–3]. It exposed critical gaps in health system preparedness and revealed how deeply human health is intertwined with geopolitics, trade dependencies, and broader societal resilience [4]. The crisis made clear that health is not a narrow sectoral issue – it is foundational to the prosperity and security of communities and nations.

The pandemic also underscored the urgent need for stronger collaboration between public health and foreign policy actors, particularly in responding to transnational emergencies [5]. Political decisions on border closures, vaccine allocation, supply chains, and international aid directly shaped health outcomes. More broadly, COVID-19 unfolded within a *polycrisis* [6] – a convergence of interconnected and compounding global shocks across health, environment, economy, and security – [7,8] which severely tested the limits of national policymaking, multilateral coordination, and global solidarity.

The COVID-19 experience was not an isolated event but a signal of broader structural vulnerabilities in the global health architecture. Other recent crises have similarly tested the limits of health systems’ capacity and governance. The 2014–2016 Ebola outbreak in West Africa was characterized by delayed international coordination and inconsistent risk communication [9]. Health considerations have historically remained peripheral in global climate negotiations. While COP28’s inaugural Health Day marked a welcome shift toward recognizing the health–climate nexus, integration remains limited relative to the scale and urgency of the challenge [10]. Meanwhile, structural shifts, including the rapid adoption of artificial intelligence (AI), evolving forms of multilateralism, and the defunding or politicization of global health institutions, continue to complicate the international health landscape [11]. These converging dynamics underscore the need for strategic, coordinated action across sectors and nations.

**In this context, health diplomacy is increasingly recognized as a critical field of practice for navigating complex challenges, addressing transnational and subnational health threats, and fostering international cooperation** [12,13]. One of the earliest institutions to support this development through structured capacity-building was the Graduate Institute of Geneva’s Global Health Centre, which has delivered training in global health diplomacy since 2007 [14].

Yet consultations across the Health Diplomacy Institutional Network (HDIN), a collaboration of over 30 academic institutions across the globe [15], revealed that the field remains underdeveloped and unevenly understood. Dedicated training programs are scarce – particularly in regions such as Africa, Latin America, and the Middle East [16] – and there is a common tendency to conflate health diplomacy training with broader global health education. Moreover, no shared standard exists to define health diplomacy or to identify the core skills and knowledge professionals need to operate effectively. This contributes to persistent gaps between evidence, health priorities, and diplomatic agendas—and limits the ability of professionals across sectors to collaborate in ways that protect and advance public health during crises and beyond.

HDIN defines **health diplomacy** as *“the art and science used by governments, civil society, the private sector, and individuals to navigate political processes to improve health outcomes.”* This framing deliberately expands the concept beyond formal statecraft, traditionally understood within international relations scholarship as the conduct of relations among states in pursuit of foreign policy objectives [17], to encompass both official and informal processes operating at domestic, regional, and international levels, reflecting the increasingly multi-actor nature of contemporary diplomacy [18]. It acknowledges that contemporary health challenges increasingly require engagement by a broader range of actors and the use of multifaceted strategies to elevate health on political agendas, resolve cross-border health challenges, and strengthen systems through negotiation, influence, and collaboration [19,20]. Health diplomacy is closely linked to the governance of health institutions but is analytically distinct: while governance concerns formal structures and institutional arrangements, health diplomacy focuses on the negotiation and strategic engagement processes that shape decision-making within and across those structures. Unlike general policy advocacy, which often seeks to influence from outside formal processes, health diplomacy operates within political and diplomatic arenas where actors reconcile competing interests.

Health diplomacy, under this framing, is not the exclusive domain of ministries of foreign affairs or health. It includes a wide range of professionals, from health practitioners and officials in justice, environment, finance, and social development ministries, to representatives of multilateral institutions, civil society, the private sector, and academia. Whether negotiating climate agreements, managing regional health crises, or advocating for equitable access to healthcare, this broad coalition of actors exemplifies health diplomacy in action.

Effective health diplomacy requires the capacity to understand and engage with power relations within and between countries. Actors do not operate on equal footing – differences in political authority, economic capacity, institutional leverage, and agenda-setting power shape negotiation dynamics and outcomes. The ability to recognize and strategically navigate these asymmetries is therefore central to advancing equitable health outcomes.

A key subset of health diplomacy is **global health diplomacy**, which operates on the international stage where health intersects with foreign policy and global governance [21]. Global health diplomacy builds on established scholarship examining the relationship between global health and foreign policy and the role of diplomacy in shaping international health outcomes [22]. It has been described as “*multi-level, multi-actor negotiation processes that shape and manage the global policy environment for health*” [23] and as “*an emerging field that addresses the dual goals of improving global health and bettering international relations*.” [24] This latter definition was originally proposed for conflict-affected and low-resource environments [25].

As new and complex global risks emerge – from pandemics and AI-driven disruptions to geopolitical fragmentation and climate volatility – the demand is growing for individuals who can bridge diplomacy, politics, and public health. Building such a workforce requires a more deliberate and structured approach to training – one that is flexible, context-sensitive, and grounded in a shared understanding of the core competencies required for effective practice.

Strengthening training alone will not resolve the structural political, economic, and institutional constraints that continue to shape global health outcomes. Deep-seated power asymmetries, geopolitical tensions, and financing inequities will continue to influence decision-making processes. However, equipping professionals with the competencies to navigate these realities more strategically, ethically, and effectively can enhance the quality of engagement within such constraints and expand the space for collaborative action.

## Why a competency-based approach to health diplomacy?

Despite its growing relevance, health diplomacy remains an underdeveloped area of professional practice, with limited shared understanding and inconsistent uptake across government ministries, regional blocs, and academic institutions. Existing training opportunities are variably structured, pedagogically diverse, and unevenly distributed across regions. Consultations across the HDIN membership – comprising academic institutions and practitioners from Australia, Canada, China, Croatia, Guinea-Bissau, Japan, Rwanda, Sweden, Switzerland, the United Kingdom, and the United States actively engaged in delivering health diplomacy training – revealed a lack of consensus on core competencies, learning outcomes, and instructional design. These recurring findings across diverse institutional and geopolitical contexts point to fragmentation in curricular focus and limited articulation of shared standards. The absence of a shared skills framework contributes to inconsistent training quality and undermines the development of an effective health diplomacy workforce. This uneven development reflects not only limited training opportunities but also separate ministerial mandates and competing political priorities that constrain sustained cross-sector investment in capacity-building.

To address these challenges, HDIN developed **a globally informed, competency-based training framework through wide-ranging consultation and co-development with academic and governmental partners.** It offers a flexible foundation adaptable to diverse institutional missions, learner profiles, and regional priorities. Rather than prescribing a single model, the HDIN Training Framework takes an outcomes-based approach, outlining core knowledge and skills for health diplomacy practice while enabling institutions to design tailored programs suited to their contexts.

To support implementation, the framework is accompanied by practical tools and adaptable methodologies. It embraces pedagogical diversity across education levels and settings, aligning with international commitments to localization, health system resilience, and multisectoral collaboration. Consistent with the localization agenda, the framework supports contextual co-design and delivery by institutions operating within their own political and cultural environments. While global in scope, it is intended for national and regional application in ways that ensure local relevance while maintaining global coherence, for example, through intergenerational learning and locally informed case studies [26].

The HDIN Training Framework supports both foundational and advanced capabilities, spanning a spectrum of roles—from early-career practitioners to senior negotiators and policymakers. Its structure ensures relevance across settings, career stages, and institutional needs.

By offering a shared, collaboratively developed foundation, the HDIN Training Framework makes a timely contribution to the professionalization of health diplomacy. It enables a more coherent and strategic approach to capacity-building—one that equips professionals and institutions to address complex health threats through informed negotiation, political and power analysis, cross-sectoral coordination, and sustained global engagement.

### The competency framework: Structure and content

The HDIN Training Framework provides a **clear yet adaptable** foundation for developing training programs that address current and emerging challenges. It defines which competencies are most critical for effective practice and illustrates how these can be developed across diverse institutional and geographic settings. **Importantly, it recognizes** that these competencies are not exclusive to formal diplomatic roles but are relevant across a broad spectrum of actors, including ministries, multilateral organizations, NGOs, civil society, academia, and the private sector.

At its core, the framework is designed to support institutions in delivering high-quality, context-responsive capacity-building initiatives. It is anchored in a set of key competencies – defined as the *observable and measurable abilities essential for effective health diplomacy practice*. Each competency is further expressed through specific capabilities that reflect applied knowledge and practical performance in diverse contexts.

The framework identifies five core competencies critical to health diplomacy today:

1. ***Essential global health knowledge***
2. ***Political and power analysis and diplomacy skills***
3. ***Strategic decision-making and leadership***
4. ***Cross-cultural and interpersonal communication***
5. ***Coalition-building and advocacy***

These competencies were defined through extensive HDIN consultations and peer review and are grounded in real-world professional practice. While the listed capabilities are not explicitly mapped to a specific level of expertise, their phrasing generally aligns with *intermediate-level practice* and can be adapted to suit different stages of career development.

The table (Table 1) below summarizes the five key competencies for health diplomacy training, alongside their associated capabilities:

**Table 1:** The five key competencies.

KEY COMPETENCY	The learner/practitioner will be able to…
**Essential global health knowledge**	• **Analyze health systems, governance, and financing** mechanisms and their interlinkages with negotiation processes (e.g., bilateral, multilateral). • **Situate global health** within broader development and policy frameworks, demonstrating its relevance to wider societal goals. • Apply relevant **legal frameworks** to inform decision-making and address complex health diplomacy challenges. • Assess contemporary global health challenges (e.g., climate change, humanitarian crises, geopolitical tensions) and their implications for diplomacy and systems resilience.
**Political and power analysis and diplomacy skills**	• Navigate formal and informal political structures, processes and power dynamics across contexts. • Identify and assess the power, interests, influence, and structural positioning of diverse stakeholders, and understand how institutional and geopolitical dynamics shape negotiation outcomes. • Reconcile domestic, regional, and international interests to support coherent foreign policy and health strategies. • Apply negotiation strategies to build consensus, resolve conflicts, and foster intersectoral collaboration. • Align health diplomacy objectives with broader political priorities to advance shared policy outcomes.
**Strategic decision-making and leadership**	• Leverage evidence and data to influence high-level policy and programmatic decisions. • Demonstrate leadership in driving innovation and managing change in dynamic environments. • Evaluate risks and make informed decisions under conditions of uncertainty. • Uphold ethical principles and values in decision-making.
**Cross-cultural and interpersonal communication**	• Cultivate trust and build relationships across diverse sectoral, geographic, and political settings. • Communicate effectively across cultural contexts, demonstrating empathy and sensitivity. • Leverage health as a platform to inform and influence political and policy-related decision-making. • Navigate communication barriers and adapt messaging to engage diverse audiences effectively.
**Coalition-building and advocacy**	• Mobilize and sustain multi-stakeholder partnerships, including with governments, multilateral agencies, communities, and civil society. • Engage constructively with the private sector while recognizing and managing potential conflicts of interest and ethical considerations. • Identify strategic opportunities for advocacy and tailor messaging to diverse audiences. • Adapt advocacy approaches to suit varied political, institutional, and cultural contexts.

## From concept to practice: Applying the framework

The HDIN Training Framework is not intended as a prescriptive curriculum. Rather, it offers a flexible and practical resource that institutions can adapt to their specific mandates, balancing global coherence with local relevance. It supports diverse applications across educational formats, political systems, institutional goals, and learner profiles.

Each competency is articulated across four progressive levels of mastery. These levels reflect increasing depth of responsibility and complexity in practice, and are designed to support curriculum development, learning objectives, and assessment strategies. Institutions can align training content to learners’ professional experience and current or anticipated roles. Importantly, the way each competency is applied will vary depending on political context, institutional mandate, and level of authority. While the core competencies provide a shared foundation, their operationalization differs across settings – for example, between national ministries, regional bodies, and multilateral forums. The progressive levels outlined below capture increasing responsibility and influence, while remaining adaptable to diverse institutional and political environments.

- ***Basic***: Demonstrates foundational knowledge and performs tasks under guidance in supervised contexts.
- ***Intermediate***: Applies knowledge and skills independently in familiar environments to achieve defined objectives.
- ***Proficient***: Leads initiatives and delivers results in complex or high-stakes situations, navigating uncertainty with confidence.
- ***Expert***: Shapes policy, strategies, and global frameworks; mentors others; and drives innovation across systems and institutions.

This progressive model supports the design of multi-tiered training programs – including workshops, executive courses, and summer schools – ensuring that learners are supported and challenged according to their career stage and institutional responsibilities.

To further support practical implementation, the HDIN Training Framework is accompanied by a suite of tools designed to help institutions build context-responsive, engaging, and outcomes-oriented curricula. **Annex A** presents examples of how competencies and mastery levels can be mapped to common health diplomacy roles – from early-career officers to senior policymakers and negotiators. **Annex B** offers a flexible menu of teaching methodologies aligned to each competency, including role-play simulations, negotiation labs, case-based discussions, policy brief development, and emerging applications of AI in both learning and practice [27].

**Importantly,** this framework complements existing capacity-building efforts in global health – such as the World Health Organization’s technical training on global governance, and UNICEF’s multisectoral policy development programs [28,29]. While many of these initiatives are thematic or agency-specific, the HDIN Training Framework provides a cross-cutting, competency-based foundation that promotes coherence across institutions and systems. Its flexible structure enables integration into national training institutes, incorporation within postgraduate public health programs, and application by multilateral agencies seeking to strengthen their staff’s skills in health diplomacy and negotiation.

### Implications and relevance

The HDIN competency-based framework for health diplomacy holds broad relevance for institutions and actors working across health, foreign policy, international development, humanitarian response, and related fields. It responds to a growing demand for professionals capable of operating at the intersection of health and diplomacy – navigating complex systems, aligning diverse interests, and advancing coherent responses to shared challenges. This demand extends well beyond responding to epidemics or pandemics to encompass climate negotiations, humanitarian crises, health service provision in conflict zones, and cross-border policy processes that increasingly shape health outcomes.

By offering a structured yet adaptable foundation, the HDIN Training Framework contributes to the professionalization of health diplomacy as a distinct but interdisciplinary field. Its design helps bridge persistent silos between health and foreign policy domains, and between technical expertise and political negotiation. Crucially, it provides a practical basis for developing programs that are pedagogically robust and tailored to institutional mandates, regional contexts, and learner profiles.

Several core features of the framework are particularly valuable for those seeking to develop or strengthen health diplomacy training initiatives:

#### 1. Competency-based structure.

A shared reference point that enhances the quality, coherence, and comparability of training programs across institutions and regions.

#### 2. Five core competencies.

A clearly defined set of skills areas critical for effective practice: essential global health knowledge, political analysis and diplomacy skills, strategic decision-making and leadership, cross-cultural and interpersonal communication, and coalition-building and advocacy.

#### 3. Progressive mastery levels.

Each competency is articulated across four levels – basic, intermediate, proficient, and expert –enabling alignment with learners’ experience and responsibilities.

#### 4. Flexible program design.

A structure that supports customization to reflect geopolitical realities, institutional priorities, and diverse learner profiles.

#### 5. Practical implementation tools.

A set of applied methodologies and role-based applications that connect the framework to real-world challenges such as pandemics, climate change, armed conflict, and geopolitical disruption.

Together, these features support the development of both individual capacities and institutional capacity, ultimately strengthening the coherence, responsiveness, and resilience of health systems. As the global health landscape becomes increasingly complex and contested, the HDIN Training Framework offers a timely and practical contribution. It helps ensure that health remains a coherent, credible, and strategically positioned component of domestic, regional, and global policy agendas.

## Conclusion: A practical foundation for action

The HDIN Training Framework offers a strategic and actionable response to the growing need for structured, high-quality training in health diplomacy. As institutions across sectors adopt and adapt this framework, it has the potential to cultivate a globally competent, ethically grounded, and politically astute workforce capable of advancing health priorities within contested policy environments.

Engagement in health diplomacy requires more than technical expertise or ad hoc exposure. It demands deliberate, context-responsive capacity-building grounded in real-world complexity and principled decision-making.

By emphasizing practical competencies rather than prescriptive curricula, the HDIN Training Framework helps bridge enduring divides between public health, diplomacy, and international policy. It provides a coherent foundation for strengthening both individual capability and institutional resilience.

In an era of intensifying geopolitical fragmentation, climate shocks, technological disruption, and persistent inequities, strengthening the political, analytical, and relational capacities of those shaping health-related policy and decision-making across sectors will be essential to advancing equitable health outcomes. The HDIN Training Framework offers a timely and pragmatic contribution toward that goal.

## Annex A: Key competencies for health diplomacy in practice

This annex illustrates how the core competencies of the HDIN Training Framework can be applied across four progressive levels of mastery (Table 2):

- ***Basic***: Demonstrates foundational knowledge and supports activities under guidance in supervised contexts.
- ***Intermediate***: Applies knowledge and skills independently in familiar environments to achieve defined objectives.
- ***Proficient***: Leads initiatives and delivers results in complex or high-stakes situations, navigating uncertainty with confidence.
- ***Expert***: Shapes policy, strategies, and global frameworks; mentors others; and drives innovation across systems and institutions.

**Table 2:** The key competencies applied across four progressive levels of mastery.

Key Competency	Basic *e.g., Desk Officer, Junior Health Attaché*	Intermediate *e.g., Health Attaché, Technical Adviser*	Proficient *e.g., Head of Delegation to WHA, WHO Regional Coordinator*	Expert *e.g., Global Health Ambassador, Minister of Health or Foreign Affairs*
**Essential global health knowledge**	**Understands** the structure of global health systems and governance frameworks with guidance	**Applies** health systems knowledge to identify challenges and propose context- appropriate solutions aligned with political and health priorities	**Integrates** deep health system knowledge into country-level diplomatic engagement and cross-border collaboration	**Leads** reform or strengthening of global health systems; **shapes** policy at the highest levels
**Key Competency**	**Basic**	**Intermediate**	**Proficient**	**Expert**
**Political and power analysis and diplomacy skills**	**Recognizes** political and power dynamics and supports negotiations and relationship-building under guidance	**Navigates** political dynamics and decision-making processes, employing foundational negotiation skills in formal and informal settings	**Leads** high-level policy dialogues and diplomatic negotiations aligning health and foreign policy goals	**Shapes** international agreements and strategies; mentors others in political engagement
**Strategic decision-making and leadership**	**Contributes** to decision-making by identifying risks and applying ethical principles under supervision	**Leads** defined initiatives using data and evidence to inform choices and upholds ethical standards	**Directs** complex initiatives and adapts strategies in evolving **uncertain** contexts	**Shapes** organizational strategy in sensitive contexts; leads system-level transformation
**Cross-cultural and interpersonal communication**	**Demonstrates** awareness of cultural differences and communicates respectfully with guidance	**Communicates** effectively across cultures, adapting messaging with empathy and sensitivity	**Mediates** sensitive interactions and uses communication to foster influence and collaboration	**Leads** high-level dialogue and institutional strategies across diverse geopolitical and cultural contexts
**Coalition-building and advocacy**	**Supports** partnerships and advocacy efforts under supervision	**Builds** and sustains multi-sectoral **t**o advance shared objectives	**Leads** coalitions and advocacy initiatives in complex policy environments	**Shapes** global advocacy agendas; mobilizes collective action and long-term alliances

The table below provides illustrative examples of how each competency might be applied across health diplomacy roles. The roles presented are drawn from government positions engaged in multilateral health settings. Comparable functions exist across other sectors and institutions advancing health diplomacy, including global summits, environmental negotiations, international health partnerships, and development finance mechanisms. Skill levels are distinct from job titles. Training programs are encouraged to adapt role descriptions and competency applications to local contexts.

Importantly, training programs should incorporate an evaluation design that enables learners to self-assess their level of proficiency for each competency, including its application in professional practice. Structured peer assessment may also serve as a useful complementary tool.

## Annex B: Training methodologies and tools to develop key competencies for health diplomacy

This annex outlines training methodologies and tools that can be adapted to different learner profiles and program formats. Institutions are encouraged to tailor these approaches based on available resources and contextual priorities.

Intended as a living resource, this annex recognizes that training methodologies and tools may evolve over time, with each contributing to multiple educational goals. The first section presents the five key competencies, each introduced with a brief overview of recommended methodologies, followed by a table of adaptable learning tools. The second section offers a glossary of key terms to support consistent understanding across institutions and regions. Emerging tools, such as AI-supported simulations or writing assistants, may be incorporated to enhance learning and adaptability, provided they are used with appropriate ethical safeguards, including attention to data integrity, bias, and confidentiality.

### Essential global health knowledge

*This key competency is best developed through structured analysis of curated materials, reinforced by group discussion and applied learning* (Table 3).

**Table 3:** Essential global health knowledge – learning objectives and tools.

The learner/practitioner will be able to	Learning Tools
• **Analyze health systems, governance, and financing** mechanisms and their interlinkages with negotiation processes (e.g., bilateral, multilateral). • **Situate global health** within broader development and policy frameworks, demonstrating its relevance to wider societal goals. • Apply relevant **legal frameworks** to inform decision-making and address complex health diplomacy challenges. • Assess contemporary global health challenges (e.g., climate change, humanitarian crises, geopolitical tensions) and their implications for diplomacy and systems resilience.	• Lectures (live or recorded) by senior leaders or experienced delegates. • **Case studies** – based on learners’ case examples in longer programs. • Pre-reading assignments or curated video materials. • **Group exercises.** • Scenario-based policy simulations • Collaborative research or group project (in longer training formats). • Site visits or case analysis of health programs in conflict settings. • Foresight exercises (for executive and master’s-level learners).

Content should be adapted to learners’ existing knowledge. For example, those with limited prior experience may focus on global health actors and domestic legislation, while more advanced learners can explore systems thinking, global health governance, and legal frameworks such as international humanitarian law and international human rights law.

### Political and power analysis and diplomacy skills

*This key competency is best developed through experiential and intergenerational learning, with an emphasis on structured practice, peer engagement, and reflective storytelling* (Table 4).

**Table 4:** Political and power analysis and diplomacy skills– learning objectives and tools.

The learner/practitioner will be able to	Learning Tools
• Navigate formal and informal political structures, processes and power dynamics across contexts. • Identify and assess the interest and influence of diverse stakeholders in health-related negotiations. • Reconcile domestic, regional, international interests to support coherent foreign policy and health strategies. • Apply negotiation strategies to build consensus, resolve conflicts, and foster intersectoral collaboration. • Align health diplomacy objectives with broader political priorities to advance shared policy outcomes.	• Experiential learning: simulations, mock negotiations and resolution or MoU drafting • Reflective storytelling by experienced practitioners. • Lectures on political theory and how health features in foreign policy. • Readings of classical texts on health diplomacy. • Curated video clips of health-related diplomatic or negotiation scenarios. • Recording and feedback on learner performance (particularly useful for early-career professionals).

Training should be tailored to learners’ level of experience and the duration of the program. For example, undergraduate learners may benefit from a focus on leadership types and power structures (the “what”), while executive-level training should emphasize applied methods such as simulation exercises and story-based reflection (the “how”). In short-format offerings (e.g., masterclasses or executive sessions), priority should be given to practical exercises.

### Strategic decision-making and leadership

*This key competency is best developed through experiential and intergenerational learning with an emphasis on applying leadership frameworks to real-world complexity* (Table 5)*.*

**Table 5:** Essential global health knowledge – learning objectives and tools.

The learner/practitioner will be able to	Learning Tools
• Demonstrate leadership in driving innovation and managing change in dynamic environments. • Evaluate risks and make informed decisions under conditions of uncertainty. • Uphold ethical principles and values in decision-making. • Leverage evidence and data to influence high-level policy and programmatic decisions.	• Simulations of strategic or crisis decision-making. • Leadership case studies involving ethical dilemmas. • Strategic and emergency planning simulations using limited, ambiguous, or conflicting information. • Facilitated debriefs to reflect on decision-making processes and extract lessons.

Content should be matched to learners’ professional experience and decision-making responsibilities. For example, those newer to the field may benefit from exploring leadership principles and strategic frameworks, while more experienced learners can focus on systems thinking, policy design, and high-stakes simulations such as crisis leadership or emergency scenario planning.

### Cross-cultural and interpersonal communication

*This key competency is developed most effectively through experiential and peer learning, with a focus on role play and group work* (Table 6).

**Table 6:** Cross-cultural and Interpersonal Communication– learning objectives and tools.

The learner/practitioner will be able to	Learning Tools
• Cultivate trust and build relationships across diverse sectoral, geographic, and political settings. • Communicate effectively across cultural contexts, demonstrating empathy and sensitivity. • Leverage health as a platform to inform and influence political and policy-related decision-making. • Navigate communication barriers and adapt messaging to engage diverse audiences effectively.	• Intercultural dialogue workshops. • Role plays focused on cross-cultural scenarios and interpersonal negotiation. • Debrief sessions to examine communication styles and cultural dynamics in case studies. • Personal storytelling. • Structured peer learning.

Activities should be adapted to learners’ communication experience and cultural awareness. For those earlier in their careers, training might focus on listening skills, non-verbal cues, and basic protocol, while more experienced learners may benefit from working on facilitation, negotiation across cultures, and managing communication in politically sensitive environments, including recognizing how language and framing can unintentionally erode trust or reinforce power asymmetries.

### Coalition-building and advocacy

*This key competency is best developed through experiential and intergenerational learning, particularly via hands-on tools such as stakeholder mapping, message framing, and simulated advocacy exercises* (Table 7).

**Table 7:** Coalition-building and Advocacy – learning objectives and tools.

The learner/practitioner will be able to	Learning Tools
• Mobilize and sustain multi-stakeholder partnerships, including with governments, multilateral agencies, communities, and civil society. • Engage constructively with the private sector to identify and support shared health and political objectives. • Identify strategic opportunities for advocacy and tailor messaging to diverse audiences. • Adapt advocacy approaches to suit varied political, institutional, and cultural contexts.	• Stakeholder and influence pathway mapping exercises. • Coalition-building role play. • Workshops on message design, influence strategies, and audience targeting. • Public speaking and message delivery sessions. • Reflective storytelling by experienced communicators. • Peer learning based on lived advocacy experience.

Training should be adapted to learners’ experience level. For example, undergraduates may begin with basic trust-building and communication strategies, while postgraduate and executive learners can explore complex coalition dynamics through advanced simulations.

## Glossary of Terms

This glossary provides brief definitions of selected learning methodologies and tools used in health diplomacy training. It aims to ensure clarity and consistency across institutions delivering programs aligned with the HDIN Training Framework.

**Case Study:** a real or hypothetical example used to analyze and discuss specific challenges, decisions, or outcomes. It can be used to teach policy analysis, strategic planning, or stakeholder engagement.

**Debriefing:** a reflective discussion that follows an exercise or activity, allowing participants to analyze what occurred, extract lessons, and connect them to theory or practice.

**Experiential Learning: a** process where learners gain knowledge and skills through direct experience. This includes simulations, field exercises, or case-based problem-solving, followed by structured reflection.

**Intergenerational Learning:** an approach that encourages knowledge exchange between individuals from different generations or career stages, often combining the experience of senior professionals with the perspectives of early-career professionals.

**Mock Exercise:** a large-scale, scenario-based activity designed to simulate a high-stakes event (e.g., multilateral negotiation or public health emergency). It usually follows a formal process and often includes structured observation, feedback, and debriefing.

**Peer Learning: a**n approach where participants learn from each other’s experiences and insights, often through discussions, group tasks, or joint problem-solving activities. It promotes collaboration, mutual feedback, and shared reflection.

**Reflective Storytelling:** a learning method where personal or professional experiences are shared to illustrate complex ideas, build empathy, or enhance understanding of historical, diplomatic, and political contexts.

**Role Play:** a method where participants assume specific roles (e.g., diplomat, health adviser) in a fictional scenario to practice communication, negotiation, or leadership skills. It focuses more on interaction and character-driven situations than simulations.

**Simulation:** a structured activity that imitates real-life scenarios to allow participants to practice skills or decision-making in a safe environment. Commonly used in diplomatic negotiations or emergency response training. Emerging AI tools may support simulations by generating dynamic scenarios, adapting role content, or providing structured feedback.

**Structured Practice:** planned, repetitive exercises designed to build specific skills (e.g., speaking, writing, negotiation), often with feedback from facilitators or peers.
